# Role of female community health volunteers for visceral leishmaniasis detection and vector surveillance in Nepal

**DOI:** 10.15171/hpp.2020.09

**Published:** 2020-01-28

**Authors:** Mazin Omer, Axel Kroeger, Anand Ballabh Joshi, Murari Lal Das, Lina Ghassan Younis, Vivek Kumar Singh, Chitra Kumar Gurung, Megha Raj Banjara

**Affiliations:** ^1^Public Health and Infectious Disease Research Center, New Baneshwor, Kathmandu, Nepal; ^2^Freiburg University, Freiburg, Germany, WHO Special Programme for Research and Training in Tropical Diseases (WHOTDR), Geneva, Switzerland; ^3^Central Department of Microbiology, Tribhuvan University, Kirtipur, Kathmandu, Nepal

**Keywords:** Female community health volunteers, Vector surveillance, Visceral leishmaniasis, Elimination

## Abstract

**Background:** As visceral leishmaniasis (VL) has recently expanded in previously non-endemic areas of Nepal, the health system is facing new challenges. Female community health volunteers(FCHVs) are playing an important role for VL elimination in Nepal. This study aimed to analyze the actual and potential role of FCHVs for VL elimination program as well as community awareness of the disease (VL) and protective measures.

**Methods:** We used a concurrent embedded mixed methods design. Qualitative data were collected through in-depth interviews and focus group discussions with FCHVs of 22 VLendemic villages of 3 districts. Concurrently quantitative data were collected through formal interviews of 203 household heads of the same villages.

**Results:** FCHVs are able to perform their duties in an efficient way with the support of their families and specific incentives. FCHVs in the VL-endemic region have a good ability to recognize the VL suspects and refer to health facilities. The feedback by the district health office on referred patients was weak thus missing the opportunity of involving FCHVs in the 6-months follow up. In houses with a previous VL case knowledge levels of prevention and treatment ofVL were significantly better than in houses without a previous VL case. More people in houses with a former VL patient were aware on VL transmission.

**Conclusion:** FCHVs are playing an important role for VL elimination in Nepal through detection of suspected cases and referral and may play a role in vector surveillance.

## Introduction

Infectious diseases cause more than 25% of the global disease toll.^1^ Visceral leishmaniasis (VL) also called Kala-azar (KA) is ranked second in mortality and fourth in morbidity among Neglected-tropical diseases “NTDs”, with 20 000 to 40 000 deaths annually.^2^ Moreover, VL is estimated to cause the ninth largest disease burden among infectious diseases and worldwide 98 countries are endemic for VL.^1,3^ The World Health Assembly 43.18 resolution has recognized leishmaniasis as a major public health concern.^4^

The highest global rate of occurrence of VL worldwide is on the Indian subcontinent with approximately 67% of all human cases occurring in India, Bangladesh and Nepal in areas of extreme poverty and high population density.^5^ VL was prevalent in Nepal but the number of cases was reduced significantly due to extensive use of DDT in the 1960s. In 2005, the governments of India, Bangladesh and Nepal launched a regional initiative to eliminate VL by the year 2015.^6^ Nepal has reached the <1 case per 10 000 elimination target with only 150 cases reported in Nepal in 2016.^7^ Nevertheless, the elimination efforts are challenged by a new geographical expansion of the disease into previously not affected areas,in which the primary health care system is having recently an increasing role in the detection of those cases.^8,9^

The primary health care system is contributing considerably to the fight against communicable diseases. One of the important aspects to ensure effective and sustainable primary health services is the participation of the community. Improved interaction among health system, people, and health volunteers, as well as training programs for and by health volunteers lead to a better utilization of society sources in health programs.^10^ The community health worker (CHW) model has been successfully used to promote health and reduce adverse health outcomes in under-served communities.^11^ Use of CHWs in healthcare provision is increasing worldwide.^12^ While some CHW programs are small independent projects, others are large nationwide programs managed by government agencies, such as the female community health volunteers (FCHVs) in Nepal. FCHVs are the lowest level or ‘first contact’ primary healthcare providers within the public healthcare system.^13^

FCHVs are typically local women above 25 years of age who receive a basic 18 days of training in various primary healthcare topics, including the fight against communicable diseases.^14^ The Government of Nepal initiated the FCHV Program in the years 1988/1989in 27 districts and expanded it to all 75 districts of the country in a phased manner.^15^ Altogether there are approximately 51 470 FCHVs in the country (47 328 FCHVs at rural level and 4142 at urban/municipality level).^7^

The major role of the FCHV is to advocate healthy behaviors of mothers and community people and to provide community health services with a focus on immunizations, vitamin A supplementation, and maternal-child health.^15^ FCHVs in Nepal were supposed to be a main contributor to the VL elimination program due to their role in the early detection of cases in the villages and their potential role in vector control. Those roles are considered relatively new in comparison to the other activities. The detection of cases is mainly through the discovering of the fever cases and referring them to the health facilities for further investigation. Vector control is implemented by vector control staff but there are recommendations to involve the Primary Health Care system for instance by collecting vectors using “the sticky paper method”, which is considered a simple and efficient method for vector surveillance.

The present role of FCHVs in VL control has not been identified and their potential role for vector surveillance has not been assessed. FCHVs can be employed in VL elimination identifying their capacities and training needs. Therefore, the aim of this study was to analyze the actual and potential role of FCHVs during the maintenance phase of the VL elimination program, in particular by assessing their workload and its relation to their performance, their ability to detect the fever cases which are suspected for VL, and refer them to the health facilities as well as peoples’ view of FCHVs and their current knowledge and understanding of the disease and transmission.

## Materials and Methods

The study used a “concurrent embedded mixed methods approach” where quantitative and qualitative data were collected simultaneously but the primary method which guided the approach was qualitative.^16^ The quantitative approach was used for the second objective only (see below). Qualitative data were collected from FCHVs to get an insight into their daily work and challenges when adding a new component (VL case detection, referral of suspected cases and treatment follow-up). Quantitative data were collected through an interview survey with household heads to get information about their awareness of the disease and preventive measures.

**Figure 1:**
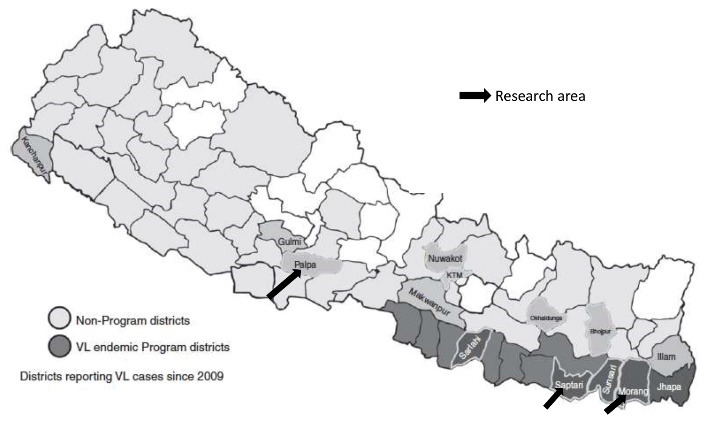
Map of program and non- program areas in Nepal highlighting the study districts.

The study was conducted during June-August 2018 in 22 VL endemic villages in three districts of Nepal in which the program of FCHVrs is implemented. Part of the research was conducted in the remote “hilly” area of “Palpa district” where VL is a new disease and the other part was conducted in the plain “Terai” region in “Saptari and Morang districts” where VL is endemic for a long time (see Figure 1). The selection of villages within the districts was based on the occurrence of VL cases during the preceding 3 to 5 years.

### Qualitative data collection

*Study design*

Qualitative data were collected from the FCHVs through face-to-face in-depth interviews at different villages within the study area. Also Focus Group Discussions (FGDs) were conducted among the FCHVs during their monthly meetings. This information was collected to identify the actual and potential role of FCHVs in the VL elimination programme.

*Participants*

We contacted 18 FCHVs in the selected villages with documented VL cases for the in-depth interviews until the saturation level was reached (i.e. no new information could be obtained). Two FGDs were performed in the Terai region. One of them was with 4 FCHVs and the other with 5 FCHVs. There was no opportunity in the hilly district to bring several FCHVs together for an FGD.

*Data collection*

The qualitative in-depth interviews were conducted usually in the houses of the FCHVs using a standard list of guiding (open-ended) questions. The list of questions was piloted, modified and translated into Nepali. The in-depth interviews with the FCHVs lasted on average one hour and were audio recorded and transcribed. Interviews were conducted in Nepali.

The FGDs were conducted during the monthly meetings of the FCHVs. The size of each group ranged from 3 to 5 individuals. The length of the discussion sessions was between half to 1 hour. The researcher was the moderator who led the discussion and there was a translator who translated and facilitated the discussion. The discussions took place in a setting where the session could not be interrupted. Each of the FGDs was audiotaped and transcribed.

*Data analysis*

All interviews and FGDs were transcribed. Thematic analysis was used for the analysis of the data, following Clarke and Brauns’s six phases of thematic analysis; familiarization, coding, searching for themes, reviewing themes, defining and naming themes and writing up.^17^

Following the transcription of the data, the investigator with the translator listened to the recorded interviews a number of times to make sure the accuracy of the transcription. In the second step, initial coding of the transcribed data was done. Then, all the similar codes were put under one final code, which gave the final categories for the analysis. According to these categories all the collected data were rearranged and organized in thematic groups.

### Quantitative data collection

*Study design*

A cross-sectional survey was conducted in the same villages as described above to collect quantitative data by applying a questionnaire to household heads in houses with or without a previous VL case. The questions were about people’s awareness, knowledge and perceptions regarding VL as well as about, protective measures against VL transmission.

*Participants*

Two hundred and three randomly selected houses (by systematic sampling) were included for the quantitative data collection, roughly 10 houses per village; 66 of them were houses with VL patients in the preceding 5 years (“cases”) and 137 houses were controls without VL patients during the preceding 5 years. For this, sample size was calculated based on case control study design using 95% CI, 80% power and estimated odds ratio of 2.

*Data analysis*

Quantitative data were entered into SPSS version 21.0 and analyzed. In descriptive analysis frequency and percentages were calculated. Odds ratio was calculated and chi-square test was used when comparing knowledge between households with VL case and households without VL case. *P* value less than 0.05 was considered statistically significant.

## Results

### Enabling factors for the FCHVs in accomplishing their duties in the community

More than 50% of the FCHVs have been working for more than 20 years. This experience is reflected in the way they set their priorities between the household tasks and the community service and at the same time the priorities among the different activities in the community services. It also shows their interest in serving their communities.

For the majority of the 18 FCHVs interviewed, the family is playing a crucial role not only to create a balance between their household tasks and their duties as FCHVs, but also in the completion and in the fulfilling of their tasks in the community services which include writing the monthly report as well as the documentation of different activities like vaccination and distributing vitamins; family member also helped with the transportation to the far-away houses of the villages.

Moreover, family members are supporting the FCHVs in distributing information about the different activities among the villagers like for example the “immunization day”.

*“The family is helping me in distributing the vaccines and vitamins. Sometimes a lot of visitors and sick people come seeking for advice in the evening, and my family helps in giving information to them. My husband in particular helps me in performing some of these duties”* (FCHV, Palpa district).

FCHVs spend considerable time in preparing the monthly “mother group meeting”, where they educate and inform the women of the village about different health issues.

FCHVs receive incentives for accomplishing some tasks like attending training programs, completion of vaccination programs, distributing some medications in the community, and also for excellence and hardworking.

*“I am getting every month 1000 rupees (8.80 $) from the district level, in addition we get for example in one day training program for some diseases like filariasis 400 rupees (3.52 $) and then 2400 rupees (21.12 $) for 2 FCHVs from the health facility to distribute the medication in the village”* (FCHV, Saptari district).

Those incentives come from different sources like from the district level, “Village Development Committees” and international organizations and includes money, certificates or household materials.

Some of the FCHVs complained that those incentives do not come in a regular pattern and sometimes they have to wait for several months in order to get a training program or receive some incentives. Moreover, most of them do not get any kind of incentives upon the reporting of a suspected kala-azar case.

### FCHVs knowledge of VL (kala-azar), early response to symptoms and follow-up after treatment (qualitative study)

All of the FCHVs interviewed in the “Terai” plain region had some training in the detection of suspected VL cases but in the “hilly” areas they were not yet trained. The majority of the FCHVs showed a very good knowledge concerning the symptoms of kala-azar; most of them could mention 3-4 symptoms of the disease, in which the fever was considered to be a constant main symptom.

Early detection of the suspected cases as well as educating the villagers about the disease were frequently mentioned tasks by the FCHVs. Most of the fever cases were discovered during the routine visits of the FCHVs to the households.

*“One problem that I face, people don’t come to me immediately when they have the symptoms, that I can refer them to the health facility, that’s why it is very important to educate them, so they could come early to me”* (FCHV, Saptari district).

Due to the lack of money for transportation to the health facilities, a suggestion from the FCHVs was to perform the diagnosis in the villages through a remote team from the district level.

*“Important for protection is to provide education to the villagers as well as referring them to the health facility as fast as possible and it would be much better if we could get the examination here in the village* ” (FCHV, Morang district).

At the same time, there was an agreement among the FCHVs, that the next step after noticing fever and the other symptoms of kala-azar in a patient was to transfer him/her directly to the health facility as soon as possible.

*“ Yes I know kala-azar as there were some cases in my area. The symptoms are fever, loss of weight, loss of appetite and fatigue, and if I have a suspected person in the village, I will refer him as soon as possible to the health facility”* (FCHV, Morang district).

Some FCHVs even suggested to accompany the suspected person to the health facility personally, to be sure that this person will get as soon as possible the diagnosis and the treatment if needed. The need for early referral is reflected in the reported actions by FCHVs: 11 (61%) of the 18 FCHVs during the last 5 years and 7 during the last 6 months had referred one or more villagers with fever and the suspicion of kala-azar to the health facilities for further check-up.

**Figure 2:**
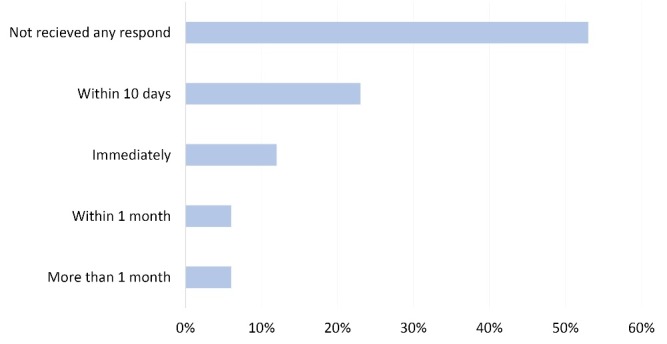
Time of response by district health team to respond to FCHVs after referring a kala-azar patient (n=18).

After the reporting of suspected cases to the health facility at district level, half of the FCHVs have not received any message back from them, as shown in Figure 2.

Regarding the follow-up of recently treated patients when they come back to their communities, FCHVs are not properly informed by the district hospital or public health office. However, sometimes it seems that there is an informal and unstructured follow up of patients when they return after treatment to their communities; FCHVs follow up when they get the information from the patient.

*“Most of the time we are informed about the diagnosis from the patient himself, mostly when we meet him/her at the market, then we follow him/her up for 10-15 days after informing us”* (FCHV, Morang district).

### Knowledge of FCHVs regarding VL transmission and prevention

Sixteen of the 18 FCHVs knew that the disease is transmitted through an “insect” (87.5%). Eleven of them knew about the sand fly (61%), others assumed that it could be a mosquito or other kind of insects.

*“The disease is transmitted from an infected person to a healthy one through the bite of the sand-fly. But I haven’t seen them before”* (FCHV, Palpa district).

Regarding preventive measures against VL transmission FCHVs mentioned among others: Preventing the accumulation of water in open spaces; maintaining adequate ventilation in the houses as well as using personal protection like mosquito nets, oils and creams against mosquito bites and usage of insecticides.

*“I think the usage of mosquito nets is very important, maintenance of cleaning and the usage of oil in the body are important factors as well to prevent the transmission of the disease”* (FCHV of Saptari district).

### Difficulties which face FCHVs in performing their tasks regarding VL (kala-azar) in their communities

The majority of the interviewed FCHVs complained about the lack of sufficient training on kala-azar; in the “hilly” region they did not receive any training about kala-azar and in the “Terai” plain region there is a deficiency in the training program. At the same time they mentioned, that the amount of money, which they receive from the district level, as well as the incentives for the different activities like reporting of the suspected cases were not enough.

*“ Yes we had some training for kala-azar, it was just a lecture for one hour in the blackboard, and we think it was not enough…we need more training if it’s possible”* (FCHV, Saptari district).

**Figure 3:**
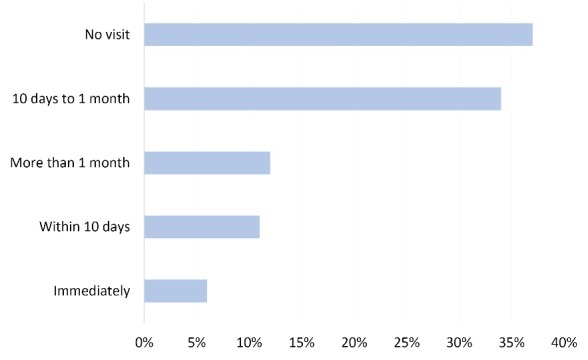
Delay in days of receiving a visit from the district office after reporting a VL case (n=18).

A “communication gap” between the FCHVs and the officials in the health facilities as well as in the district health office seems to be one of the major difficulties that face the FCHVs. Figure 3 shows an approximation of the travel time needed from the district level to visit the villages after receiving the message from the FCHVs about a suspected case. Another aspect is the deficient infrastructure, which connects the villages with the central level.

The low educational level of the villagers, which leads to the refusal of the confirmatory diagnosis, is considered by FCHVs to be a challenge. On the other hand, the financial situation of the low-income villagers is also playing an important role in terms of lack resources for the transport to the health facility as well as for the treatment (although the VL treatment itself is free of charge).

Another difficulty which faces the FCHVs is that they do not know about the results of the confirmatory investigation neither from the health facility nor from the patient, which makes it more difficult for them to follow-up the patient after the confirmation of the disease.

*“Sometimes the suspected cases of kala-azar with the fever are hesitating in going to the health facility either because they don’t have money or do not believe in the disease, that’s why sometimes we have to accompany them personally to the health facility”* (FCHV, Morang district).

### People’s awareness of VL as a key for early case detection

**Figure 4:**
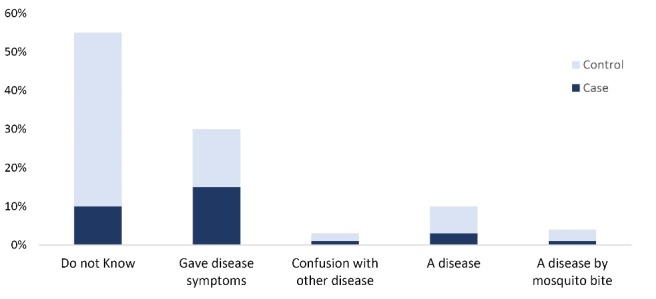
Interviewees response to “What is kala-azar?” (n = 203).

The formal interviews in households with and without previous VL cases were conducted to understand the level of peoples` awareness and concerns regarding VL disease in their communities as the basis for prevention (mainly protection from vector biting) and early self-diagnosis and seeking care. It was found that peoples’ knowledge of kala-azar in houses with a previous VL case (“cases”) was significantly better than in houses without a previous VL case (“controls”) (see Figure 4, OR 3.056, *P* = 0.000).

**Figure 5:**
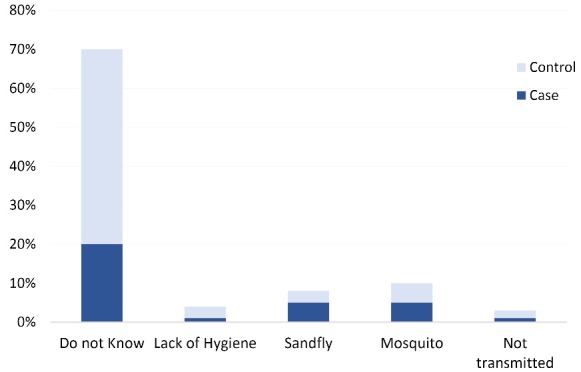
Answers to “How is kala-azar transmitted?” (n = 203).

Regarding the knowledge of the VL transmission route, more people in houses with a former VL patient were aware of the transmission route of the disease (“via insects called sandfly/housefly”, which “lives inside bedroom”), but more than 50% of all interviewees knew it is transmitted by a “mosquito”. However, 40% confounded VL with another disease, such as TB and said that respiration, food and bed sharing transmitted VL; 14.3% thought that it was a non-communicable disease (Figure 5).

When asked for personal protection, 60% of the interviewees in “control houses” did not know how they could protect themselves from VL. In contrast, former VL patients were aware that they needed to go immediately to the hospital when they have symptoms and to continue with the treatment course and follow-ups.

## Discussion

### FCHVs’ coping mechanisms with excessive workload and need for further support 

Generally, FCHVs have a high workload in terms of the tasks assigned to them by the Ministry of Health and demands by the population; they are considered to be the first contact in the villages with the primary health care system.^7,13^ Despite the high workload, they are able to perform their duties in community services often in an efficient way because of the continuous support by their families while they are conducting their community work or helping with their household activities.

There are many factors playing a role in the high workload of the FCHVs. In addition to the daily scheduled program, they are responsible for unscheduled events like emergency deliveries, sudden sickness of a villager or unplanned meetings. At the same time, they have their daily activities in their communities, which range from providing immunization, vitamins, antenatal care to organizing and leading the “mother groups”, the follow up of the treatment of sick individuals and attending meetings and training programs.^18-20^ However, some of the FCHVs prioritized their agricultural and household activities over their community obligations in view of its importance in the Nepali rural family life. But in most cases, we noticed the importance of the contribution of the family in fulfilling and completing both community and household tasks. The family members, mainly husbands, help occasionally with some activities like preparing immunization campaigns, preparation of the monthly report, and also in accomplishing the household activities; we found in several cases that the husband cleaned or cooked for the family until the FCHV finished her community task. This continued family support reflects the appreciation of the FCHVs role in the community.^21^ To perform their duties, the financial support seems to play an important role for the FCHVs, which is consistent with other data underlining the importance of incentives to sustain their work.^22,23^ Incentives for different activities like training programs help them filling the financial gaps for their families but those activities are not frequent; several months could pass without any extra incentive either from the district level, village development committee or even international and local organizations. Those extra activities could be organized in a better way throughout the year to provide a more continuous source of income to the FCHVs.

### Abilities of FCHVs to recognize and refer suspected VL cases and monitor the post-treatment phase

The FCHVs in the “Terai” plain region showed the ability not just to recognize the fever as a main symptom of VL suspected cases, but also to recognize, at least theoretically, other 2-3 symptoms of VL like darkness of the skin and swelling of the abdomen, which reflects a relatively solid knowledge of the clinical picture of the disease. This provides a basis to perform their obligation of detecting suspected cases in their localities. In contrast, the authorities in the “hilly” region of the country are just starting with the implementation of the VL program in these areas because the disease is newly introduced in that region, hence, the FCHVs in the “hilly” region did not receive any training on the recognition and the detection of VL suspected cases. Furthermore, all of the FCHVs who were trained in the detection of suspected VL cases agreed that they had and they would refer them as fast as possible to the health facility to perform the diagnostic tests. Some FCHVs claimed that they will accompany the suspected cases to the health facility and even sometimes paying the transport cost, if they felt that he/she is hesitating for any reason to go for the examination.^13^ This kind of positive attitude from the FCHVs reflects the degree of awareness that they have of the disease and the feeling of responsibility for the health status of the villagers.

If the FCHVs discover patients who returned from VL treatment they may currently perform some kind of follow-up, which usually does not exceed the period of 2 weeks but should be at least for 6 months and in a standardized way. The follow-up period is very important because of the risk of relapses, post-treatment deaths and the development of skin lesions called “post kala-azar dermal leishmaniasis” (PKDL). During this period, the patient may still be infectious and capable to transmit the parasite to healthy individuals through the sand fly. That is why it is important to train and involve FCHVs in the follow-up of VL patients after completing the treatment for 6 months.^24^

### Need to raise community awareness of VL for achieving community involvement in vector control and early treatment

One of the challenges, that the FCHVs face in the detection of suspected cases particularly in the hilly region is the low awareness level of the population about the disease which leads to the delay in detecting the cases and to a further spread of the disease. This lack of awareness was reflected in household head interviews which showed that families which had no previous experience with VL were particularly vulnerable for neglecting vector control at home and for delayed health seeking behavior as they had much lower awareness- and knowledge-levels of VL disease and the transmission route than families where a case had occurred. Ineffective teaching and learning processes, lack of health educators’ motivation, communication gaps, and lack of resources and facilities for awareness programs are possible barriers towards achieving the collaboration of rural communities with vector control and early case detection.^25^ Most of the time, the FCHVs are not called by families with a fever patient but discover the fever cases during their household-visits by chance. This delay in discovering suspected cases adds to the total delay time which includes reporting the cases and receiving an early response message from the district level (which could take from 10 days to one month) and the delay in the visit of a team from the district to the village.^26^ Therefore, a constant recommendation among the FCHVs was to provide more training sessions and information about VL, so that they could take part in scaling up the awareness level of the villagers through their different activities. The regular “mother group” meeting could be a good opportunity to inform mothers and hence, their families about the disease^13^ reflecting the importance of the FCHVs in raising the awareness of their local communities.^27,28^ Also the mutual trust among health workers of the formal health sector (health posts, local hospitals, public health offices), FCHVs and villagers should be enhanced to promote efficient health seeking behaviours among villagers.^29^ Recommendations arising from our study are presented in Supplementary file 1.

The FCHVs did not yet have received any formal training in the usage of the sticky trap method for the surveillance of the sand fly vector in their villages. The sticky paper method is considered to be simple enough to be implemented and to be collected by non-professionals like the FCHVs (unpublished document).

Involving FCHVs in VL elimination programme for referring of fever cases suspected of VL and collection of vectors through sticky traps for vector surveillance is the positive aspect since they are living in the community and their participation can be sustainable and effective. However, there are some challenges of involving them in VL elimination programme since FCHVs are un-paid volunteers and they are already occupied with several other activities such as vitamin A distribution, mother’s group meeting, referral of maternal and neonatal cases with alarm signals, distribution of family planning devices, referral of newborn for immunization. Additionally, some of the FCHVs are illiterate and may not be able to perform well as the program requires.

## Conclusion

FCHVs are playing or may play an important role in the maintenance of the elimination phase of VL in Nepal through their abilities to detect fever cases suspected for VL and refer them to the health facilities. More efforts should be spent on developing their abilities to detect the vector, so that they could contribute to the vector surveillance efforts in their villages. This study reflects FCHVs program in the primary health system as a major component in the fight against vector-borne diseases. Awareness of VL disease, treatment and transmission-prevention should be promoted through appropriate media and FCHVs and health workers in the community.

## Ethical approval

Informed consent was obtained prior to interviewing in the form of a signature after reading the consent form. All participants were informed of the nature of the study prior to their participation. In addition, the participants were informed that at any time during the interview or focus group discussion they have the freedom to refuse to answer a question or to quit the interview. All respondents were also informed that their participation is voluntary and that their responses would remain anonymous. Names were not put in the result section behind personal quotes because of data protection issues. Ethical approval was obtained from the University of Freiburg, ERC-WHO and Nepal Health Research Council.

## Competing interests

The authors declare that they have no competing interests.

## Funding

None.

## Authors’ contributions

MO: concept, study design, study tool design, data collection, data analysis, writing of the manuscript. AK: study design, study tool design, critically contributed to data analysis and the manuscript. ABJ: study design, mediation to experts for data collection, logistical support. MLD: study design, mediation to experts for data collection, logistical support. LGY: study design, data collection. VKS: mediation to experts for data collection, logistical support. CKG: study design, mediation to experts for data collection, logistical support. MRB: study design, study tool design, data analysis, critically contributed, read, and edited the manuscript. All authors read and approved the final manuscript.

## Acknowledgments

The authors would like to thank all the FCHVs, translators: for participating in the interviews, the driver allowing us to reach rural, marginalized communities and the translator. We also immensely grateful to the ASA program in Germany which has facilitated the study and to the team of the “Public Health and Infectious Disease Research Center” in Nepal for their scientific and logistic support during the field work period.

## Supplementary Materials

Supplementary file 1 contains recommendations arising from our study.
