# Supplementary material for: Role of female community health volunteers for visceral leishmaniasis detection and vector surveillance in Nepal
Source: Health Promot Perspect. 2020 Jan 28;10(1):50–8. doi: 10.15171/hpp.2020.09 (PMC7036200; doi:10.15171/hpp.2020.09)
Supplement: Supplementary file 1 [file hpp-10-50-s001.pdf]

Supplementary file 1. Recommendations

Based on the results of the study, we have the following recommendations:

- more training to the FCHVs about VL should be conducted to raise their awareness level of the disease itself and improve the impact of their work. Different strategies to scale up the educational level of the villagers should be discussed with the FCHVs during the monthly meetings as part of their learning process.
- FCHVs could contribute in the detection process of sporadic cases in the maintenance phase of VL elimination as well as in the vector surveillance procedure after receiving a theoretical as well as a practical training program about the usage of “sticky paper” method for the vector surveillance and early detection of new foci of infection.
- the extra-activities and training events should be organized more frequently throughout the year without accumulating them at a specific time of the year, to ensure a continuous learning process and a balanced distribution of the incentives throughout the year.
- the follow-up process of VL patients should be better organized and last at least for 6 months - and if possible longer- after the completion of the treatment because of the risk of relapses, post-treatment severe events and the development of the infectious “post kala azar dermal leishmaniasis (PKDL).
- the communication level between the FCHVs and the health facilities/district level should be improved and the response-time to new VL cases by the district health office should be shortened, to avoid unnecessary delays and thus the spread of the disease.
